# A revised perspective on the evolution of the lateral frontal cortex in primates

**DOI:** 10.1126/sciadv.adf9445

**Published:** 2023-05-19

**Authors:** Céline Amiez, Jérôme Sallet, Camille Giacometti, Charles Verstraete, Clémence Gandaux, Valentine Morel-Latour, Adrien Meguerditchian, Fadila Hadj-Bouziane, Suliann Ben Hamed, William D. Hopkins, Emmanuel Procyk, Charles R. E. Wilson, Michael Petrides

**Affiliations:** ^1^ Univ Lyon, Université Lyon 1, Inserm, Stem Cell and Brain Research Institute U1208, 69500 Bron, France.; ^2^ Wellcome Integrative Neuroimaging Centre, Department of Experimental Psychology, University of Oxford, Oxford OX1 3SR, UK.; ^3^ Laboratoire de Psychologie Cognitive, UMR7290, Université Aix-Marseille, CNRS, 13331 Marseille, France.; ^4^ Station de Primatologie CNRS, UPS846, 13790 Rousset, France.; ^5^ Brain and Language Research Institute, Université Aix-Marseille, CNRS, 13604 Aix-en-Provence, France.; ^6^ Integrative Multisensory Perception Action and Cognition Team (ImpAct), INSERM U1028, CNRS UMR5292, Lyon Neuroscience Research Center (CRNL), Lyon, France; University of Lyon 1, Lyon, France.; ^7^ Institut des Sciences Cognitives Marc Jeannerod, UMR5229, CNRS-Université Claude Bernard Lyon I, Bron, France.; ^8^ Department of Comparative Medicine, University of Texas MD Anderson Cancer Center, Bastrop, TX, 78602, USA.; ^9^ Department of Neurology and Neurosurgery and Department of Psychology, Montreal Neurological Institute, McGill University, Montreal, QC, Canada.

## Abstract

Detailed neuroscientific data from macaque monkeys have been essential in advancing understanding of human frontal cortex function, particularly for regions of frontal cortex without homologs in other model species. However, precise transfer of this knowledge for direct use in human applications requires an understanding of monkey to hominid homologies, particularly whether and how sulci and cytoarchitectonic regions in the frontal cortex of macaques relate to those in hominids. We combine sulcal pattern analysis with resting-state functional magnetic resonance imaging and cytoarchitectonic analysis to show that old-world monkey brains have the same principles of organization as hominid brains, with the notable exception of sulci in the frontopolar cortex. This essential comparative framework provides insights into primate brain evolution and a key tool to drive translation from invasive research in monkeys to human applications.

## Teaser

Monkeys and hominids share key principles of frontal sulcal pattern organization, providing insight into primate brain evolution.

## INTRODUCTION

The pattern and specific intersections of sulci in the cerebral cortex of the brain are reliable determinants of the anatomo-functional organization. For example, in the frontal cortex, the sulcal pattern indicates the positioning of different functional and cytoarchitectonic areas (*1*–*10*). Thus, the sulcus-determined functional anatomy is essential for interpreting neuroscientific data and for assessing clinical options in single individuals based on their specific sulcal anatomy (*11*). In many cases, interpreting group averaged functional activation data projected onto a template brain is blurred by the individual anatomical variations (*2*, *3*). For example, we have shown that the presence or absence of a paracingulate sulcus on the medial wall in human subjects has an impact on the localization of functional foci in that region of the cortex (*2*).

Much of our detailed understanding of frontal cortical function comes from invasive studies carried out in macaque monkeys. Given the importance of sulcal anatomy, it becomes critical to determine whether and how the sulci in the frontal cortex of old-world primates (macaques and baboons) compare to those of hominids (great apes and humans), yet, to date, we lack understanding of this relationship (see fig. S1 for the position in the phylogenetic tree of the study species). Describing it will provide critical information about the evolution of the anatomo-functional organization of the brain (*12*), but it is also essential if we are to use properly the plethora of neurophysiological, neuroanatomical, and neuropsychological data obtained in the past century in macaques to understand the human frontal cortex. We developed a methodology to examine morphological characteristics and variability of sulci across primates that has shed new light on the evolution of the anatomo-functional organization of the medial frontal cortex (*12*). In this comparative endeavor, the lateral portion of the prefrontal cortex has particular importance, given that its granular part is thought to be without homolog in nonprimate species (*13*–*15*). We therefore applied our methodology to provide clear identification of whether and how lateral frontal cortex from the central sulcus to the frontal pole varies across the primate order and to what extent direct cross-species homologies can be drawn. We show that old-world monkeys and hominids share key and previously unreported principles of frontal sulcal pattern organization.

**Fig. 1.**
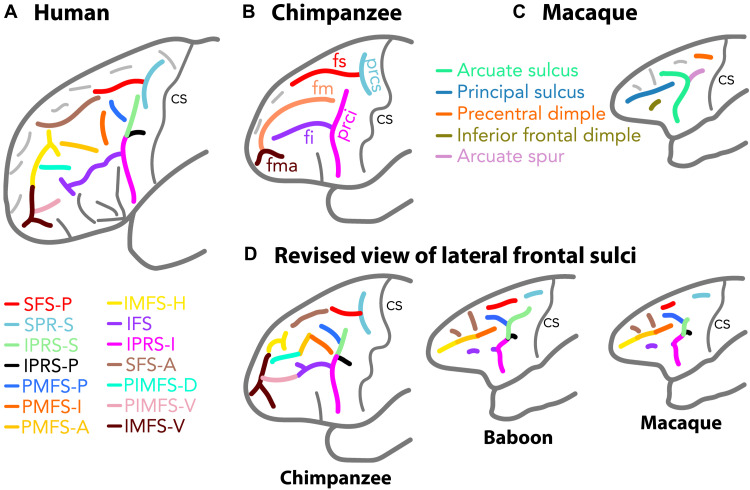
Known and revised view of the sulcal organization in the lateral frontal cortex in human, chimpanzee, baboon, and macaque brains. Known sulcal organization in the lateral frontal cortex of humans (**A**), chimpanzees (**B**), and macaques (**C**). (**D**) Revised view of the human homologs of the frontal cortical sulcal organization in the chimpanzee and old-world monkey brains. The color coding of the sulci in (D) corresponds to the sulci identified in the human brain. All sulci observed in the lateral frontal lobe of the human brain have clear homologs in chimpanzees, and only the sulci in the most anterior part of the frontopolar cortex do not have their homologs in old-world monkeys. fs and fi, superior and inferior frontal sulcus; prcs and ipcs, superior and inferior precentral sulcus; fm, middle frontal sulcus; fma, frontomarginal sulcus; SFS-P and SFS-A, posterior and anterior superior frontal sulcus; IFS, inferior frontal sulcus; SPR-S, superior precentral sulcus; IPRS-S, IPRS-P, and IPRS-I, superior, posterior, and inferior segments of the inferior precentral sulcus; PMFS-P, PMFS-I, and PMFS-A, posterior, intermediate, and anterior posteromedial frontal sulcus; IMFS-H and IMFS-V, horizontal and vertical rami of the intermediate frontal sulcus; PIMFS-D and PIMFS-V, dorsal and ventral paraintermediate frontal sulcus; cs, central sulcus. The dark gray sulci represent sulci that are located in the ventrolateral prefrontal cortex and are excluded from this analysis. The light gray sulci represent sulci that have not been named yet.

The current view of the sulcal organization of primate lateral frontal cortex is that it is characterized by 13 sulci in the human brain (Fig. 1A) (*16*); that it is restricted to five large sulci in chimpanzees (Fig. 1B) (*17*, *18*); and that, in old-world primates monkeys, it is defined by two major sulci (arcuate and principal sulcus) with up to four dimples located around these main sulci (Fig. 1, C and D) (*19*). By combining sulcal pattern analysis based on anatomical magnetic resonance imaging (MRI) (*12*) with resting-state functional MRI (rs-fMRI) and cytoarchitectonic analyses, we demonstrate that, despite apparent discrepancies, old-world monkeys share similar principles of organization to those of the chimpanzee and the human brains, with the exception of sulci associated with the rostral-most part of the frontopolar cortex that displays a similar organization only in great apes and humans. Thus, the sulci in the lateral frontal cortex of old-world monkeys can be directly understood in relation to those observed in the human frontal lobe, generating a revised framework to drive direct comparison of macaque monkey and human frontal cortical organization (Fig. 1D and Tables 1 and 2).

**Table 1.** Old world-monkeys: Correspondence between sulci defined with the traditional versus the revised nomenclature used here based on homologies with sulcal organization in the human brain.

Old nomenclature	Human-based nomenclature	
Precentral dimple	SPR-S	Superior precentral sulcus
Arcuate sulcus and spur	PMFS-P	Posterior segment of the posterior middle frontal sulcus
	IPRS-S	Superior segment of the inferior precentral sulcus
	IPRS-P	Posterior segment of the inferior precentral sulcus
	IPRS-I	Inferior segment of the inferior precentral sulcus
Principal sulcus	PMFS-I	Intermediate segment of the posterior middle frontal sulcus
	PMFS-A	Anterior segment of the posterior middle frontal sulcus
	IMFS-H	Horizontal ramus of the intermediate frontal sulcus
Inferior frontal dimple	IFS	Inferior frontal sulcus

**Table 2.** Chimpanzees: Correspondence between sulci defined with the traditional versus the revised nomenclature used here based on homologies with sulcal organization in the human brain.

Old nomenclature	Human-based nomenclature	
Superior precentral sulcus (prcs)	SPR-S	Superior precentral sulcus
Inferior precentral sulcus (prci)	IPRS-S	Superior segment of the inferior precentral sulcus
	IPRS-P	Posterior segment of the inferior precentral sulcus
	IPRS-I	Inferior segment of the inferior precentral sulcus
Superior frontal sulcus (fs)	SFS-A	Anterior superior frontal sulcus
	SFS-P	Posterior superior frontal sulcus
Inferior frontal sulcus (fi)	IFS	Inferior frontal sulcus
Middle frontal sulcus (fm)	PMFS-P	Posterior segment of the posterior middle frontal sulcus
	PMFS-I	Intermediate segment of the posterior middle frontal sulcus
	PMFS-A	Anterior posteromedial frontal sulcus
	IMFS-H	Horizontal ramus of the intermediate frontal sulcus
Frontomarginal sulcus (fma)	IMFS-V	Vertical ramus of the intermediate frontal sulcus

## RESULTS

### Hominid correspondence of the arcuate sulcus in old-world monkeys

**Fig. 2.**
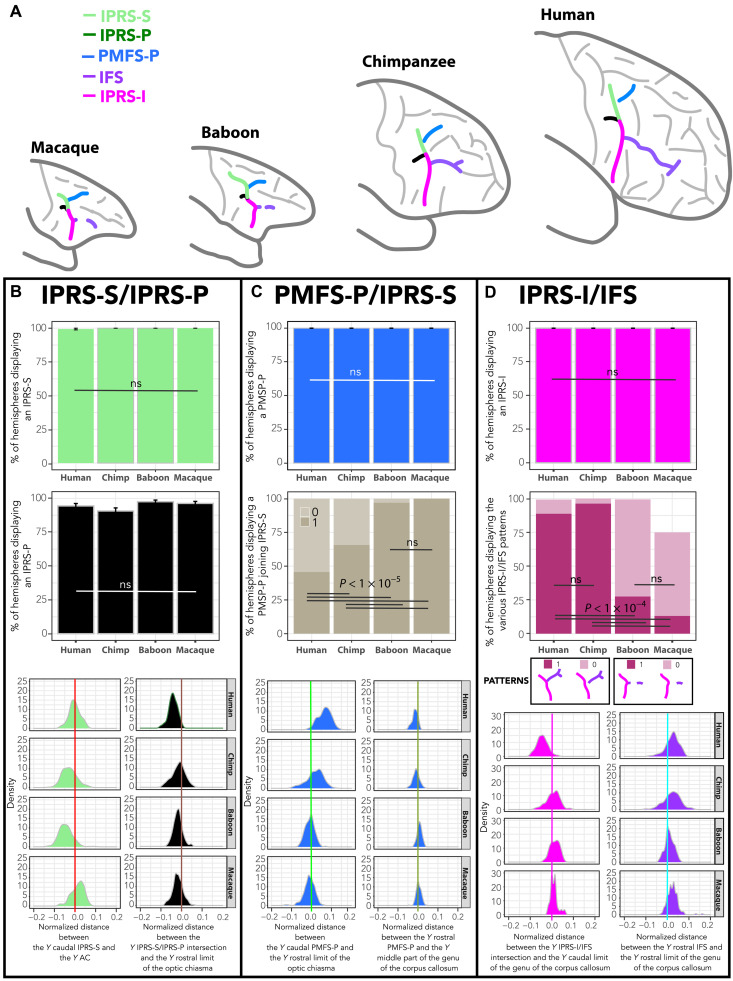
Hominid sulcal correspondence to the arcuate sulcus in old-world monkeys. (**A**) Identification and location of the sulci forming the arcuate sulcus in old-world monkeys and their homologs in hominids. (**B**) Frequency of occurrence of IPRS-S and IPRS-P in brain hemispheres in all primates (top panels) and normalized distance between the anteroposterior (i) *Y* level of the caudal-most part of IPRS-S and *Y* level of the anterior commissure (AC) (light green) and (ii) *Y* level of the intersection between the caudal-most part of PMFS-P and *Y* level of the rostral limit of the optic chiasma (dark green) (bottom panel). (**C**) Frequency of occurrence of PMFS-P (top panel) and of hemispheres displaying a PMFS-P joining IPRS-S (middle panel) in all primates, as well as normalized distance between (i) the *Y* level of the caudal-most part of PMFS-P and the *Y* level of the rostral limit of the optic chiasma (blue) and (ii) *Y* level of the rostral-most part of PMFS-P and the *Y* level of the middle part of the genu of the corpus callosum (bottom panel). (**D**) Frequency of occurrence of IPRS-I and the various IPRS-I/IFS patterns in brain hemispheres in all primates (top panels) and normalized distance between the (i) *Y* level of the IPRS-I/IFS intersection and the *Y* level of the caudal part of the genu of the corpus callosum (pink) and (ii) *Y* level of the intersection between the rostral-most part of IFS and the *Y* level of the rostral limit of the genu of the corpus callosum (purple) (bottom panel). ns, nonsignificant. GLMM and/or Tukey post hoc tests at *P* < 0.05 (see Materials and Methods).

The present research reveals the complex morphology of the arcuate sulcus in old-world monkeys (Fig. 1A). We demonstrate that the field should stop considering the arcuate sulcus as a single linear sulcus as previously thought (Fig. 2A) (*17*, *18*) but rather as a complex of sulcal elements that relate to specific sulci in hominid brains (Tables 1 and 2).

First, in old-world monkeys, below the genu of the arcuate sulcus—and the arcuate spur if present—we consistently observed the presence of an additional spur or dimple deep within this sulcal complex that had not been described previously (Fig. 2B; see typical examples and frequency of occurrence in fig. S2). The intersection between this spur/dimple and the arcuate sulcus was located at the level of the rostral limit of the optic chiasma in both macaques and baboons (see fig. S3 for description of anatomical landmarks). At this anatomical level, we identified the intersection between the superior segment of the inferior precentral sulcus (IPRS-S) and the posterior segment of the inferior precentral sulcus (IPRS-P) in chimpanzee and human brains. Furthermore, the caudal limit of the arcuate spur in old-world monkeys was observed at the level of the anterior commissure; however it is located at the caudal limit of the IPRS-S in chimpanzees and humans. Note that, in our sample, the arcuate spur was present either in the form of a spur or a dimple in 100 and 93.75% of hemispheres in baboon and macaque brains, respectively (fig. S4). The variability across studies in the frequency of occurrence of the arcuate spur in the macaque brain (*20*) is likely to be driven by the criteria used to define the presence or not of this arcuate spur, i.e., the inclusion or not of an arcuate spur with a dimple morphology. In our sample, we observe that, in macaques, the arcuate spur is observed as a spur, a dimple, or absent in 50, 44, and 6% of hemispheres, respectively (fig. S4C). Together, these data converge toward homologies between (i) the spur/dimple observed below the genu of the arcuate sulcus in old-world monkeys and the IPRS-P in chimpanzees and humans and (ii) the part of the arcuate sulcus extending from the caudal-most part of the arcuate spur to the IPRS-S/IPRS-P intersection in old-world monkeys and IPRS-S in chimpanzees and humans. The frequency of occurrence of IPRS-S and IPRS-P was identical across the four species examined {IPRS-S presence: *F* = 0.803, numerator degree of freedom (NumDF) = 3, denominator degree of freedom (DenDF) = 226.06, *P* < 0.4931 [nonsignificant (ns)]; IPRS-P presence: *F* = 2.44, NumDF = 3, DenDF = 386.38, *P* < 0.06 (ns), generalized linear mixed-effects model (GLMM) with species as fixed effect and subject ID as random effect; Fig. 2B}.

Second, the junction between IPRS-S in old-world monkeys and the part of the arcuate sulcus running dorsally and rostrally in the frontal cortex was tightly linked with the rostral part of the optic chiasma. Its rostral-most part was observed at the level of the middle part of the genu of the corpus callosum. In the human and chimpanzee brains, these two landmarks did correspond to the caudal and rostral limit of the posterior posteromedial frontal sulcus (PMFS-P), respectively. The PMFS-P is present in 100% of hemispheres in all species (Fig. 2C). It corresponds in chimpanzees to the caudal-most part of middle frontal sulcus (fm; Fig. 1B), but we observed that this sulcus most often splits into several segments, rather than being a continuous unique sulcus as previously described (*17*, *18*). Critically, we observed a gradient regarding the morphological pattern of the PMFS-P/IPRS-S junction: Whereas PMFS-P is detached from the IPRS-S in 54% of hemispheres in humans, it is detached in 34% of hemispheres in chimpanzees and in 3% in baboons, and it is never detached in macaques (*F* = 76.84, NumDF = 3, DenDF = 352.03, *P* < 2.2 × 10^−16^, GLMM; Fig. 2C and fig. S5). Our data thus strongly point toward homologies between the human PMFS-P, the caudal-most part of the chimpanzee fm, and the dorsal part of the arcuate sulcus in old-world monkeys (Fig. 1D). The arcuate spur, when present, is formed when PMFS-P joins IPRS-S anterior to IPRS-S’s caudal extremity. As such, together with evidence regarding the anatomical location of the caudal and rostral borders of IPRS-S, the arcuate spur appears to belong to IPRS-S and is not an additional sulcus (Fig. 2 and figs. S4 and S5).

Last, the ventral branch of the arcuate sulcus running below the IPRS-S/IPRS-P junction presents a dimple pointing rostrally in the frontal cortex in 27 and 13% of baboon and macaque hemispheres, respectively (Fig. 2D). The intersection between the ventral branch of the arcuate sulcus and this rostrally pointing dimple can be understood as the homolog of the junction between inferior segment of the inferior precentral sulcus (IPRS-I) and inferior frontal sulcus (IFS) in human and chimpanzee brains. First, both the ventral branch of the arcuate sulcus in old-world monkeys, the inferior part of prci in chimpanzees, and IPRS-I in humans occur in 100% of hemispheres (Fig. 2D and fig. S7). In addition, the intersection between IPRS-I/prci and IFS/fi in humans/chimpanzees is located at the level of the caudal part of the genu of the corpus callosum, exactly where the intersection between the ventral arcuate sulcus and the spur/dimple emerging rostrally is observed in old-world monkeys (Figs. 1 and 2D). This spur/dimple therefore corresponds to the precursor of the caudal part of the IFS. Topographically, in front of the ventral branch of the arcuate sulcus, only the inferior frontal dimple is observed (in 99% of baboon hemispheres and 74% of macaque hemispheres; Figs. 1A and 2D and fig. S7), and the assessment of its location revealed a tight relationship with the rostral part of the genu of the corpus callosum. However, in human and chimpanzee brains, the IFS always rostrally ends in a fork (Fig. 2D and fig. S7), which is also observed at the level of the rostral part of the genu of the corpus callosum. As such, one may hypothesize that the inferior frontal dimple is the homolog of the rostral-most part of the IFS in human and chimpanzee brains. This hypothesis finds support in our results showing that the IPRS-I/IFS pattern displays a gradient from the last common ancestor of humans and old-world monkeys to the last common ancestor of humans and apes, i.e., an IFS displaying a consistent rostral limit and rarely joining IPRS-I in old-world monkeys and a continuous sulcus displaying consistently a rostral sulcal fork and most often joining IPRS-I in hominids (*F* = 194.09, NumDF = 3, DenDF = 451.9, *P* < 2.2 × 10^−16^, GLMM; Fig. 2D). The present hypothesis is also supported by the known cytoarchitectonic organization of the region in both macaques and humans: Specifically, both the IFS and the inferior frontal dimple are the limits of area 45 (below) and area 9/46 (above) (fig. S6) (*21*, *22*).

To sum up, our results suggest that the arcuate sulcus in old-world monkeys is composed of four segments: (i) PMFS-P, the dorsal part of the arcuate sulcus that detaches progressively in the primate phylogenetic tree; (ii) IPRS-P, the newly defined little spur/dimple below the genu of the arcuate sulcus; (iii) IPRS-S, which extends from the intersection between IPRS-P and the arcuate sulcus and the caudal end of the arcuate spur; and (iv) IPRS-I, i.e., the part of the inferior arcuate sulcus located ventral to the intersection between IPRS-P and IPRS-S.

### Hominid correspondence of the supraprincipal dimples in old-world monkeys

**Fig. 3.**
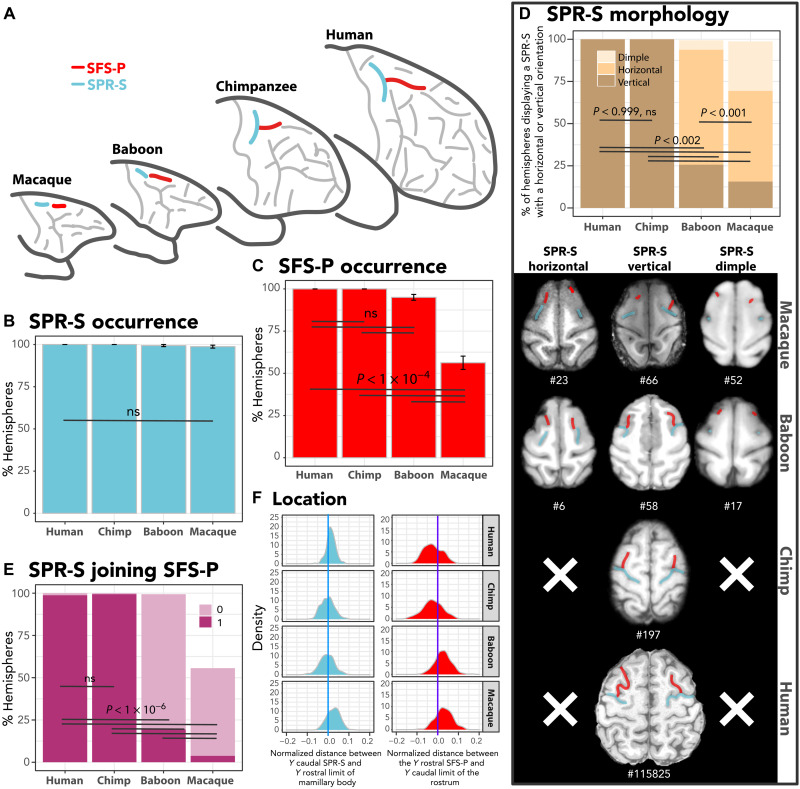
Hominid correspondence to the precentral dimples in old-world monkeys. (**A**) Identification and location of the sulci forming the precentral dimples in old-world monkeys and their homologs in hominids. Frequency of occurrence of SPR-S (**B**) and SFS-P (**C**), and (**D**) of the various orientations of SPR-S (vertically or horizontally oriented to SFS-P or nonoriented dimple) in all primates. Whereas SPR-S is equally present in all species, SFS-P displays a decreased frequency of occurrence in macaques. The hominid-specific SPR-S vertical orientation can be observed in a few hemispheres in old-world monkeys. (**E**) Percentage of hemispheres in which SPR-S joins SFS-P. Whereas the SPR-S joins SFS-P in most of the hemispheres in hominids, it is rarely the case in old-world monkeys (*F* = 528.9, NumDF = 3, DenDF = 502.38, *P* < 2.2 × 10^−16^, GLMM). (**F**) Normalized distance between the (i) *Y* level of the caudal-most part of SPR-S and the *Y* level of the rostral limit of the mamillary bodies (blue) and (ii) *Y* level of the rostral-most part of SFS-P and the *Y* level of the caudal limit of the rostrum (red). GLMM and/or Tukey post hoc tests at *P* < 0.05 (see Materials and Methods).

Previous atlases had reported the existence of up to four dimples dorsal to the arcuate and principal sulci, but their relationship to human sulci had never been formally addressed before. First, we identified correspondence between the superior precentral sulcus (SPR-S) in human (SPR-S; Fig. 1A) and chimpanzee brains (prcs; Fig. 3B) and the caudal precentral dimple in the dorsal part of the frontal lobe in macaques and baboons (Fig. 3C). Topographically, this is the first dimple in front of the central sulcus and is considered to be the border between the primary hand motor cortical region and the dorsal premotor cortex (*23*–*25*). The sulcus with similar properties is prcs in chimpanzees and SPR-S in humans (Fig. 3A). In all species, the frequency of occurrence of this sulcus/dimple is highly preserved (*F* = 1.23, NumDF = 3, DenDF = 532.64, *P* < 0.3, GLMM; Fig. 3, A and B). Second, in the four species examined, the caudal-most part of SPR-S is located at the level of the rostral limit of the mammillary bodies, and the rostral-most limit of posterior superior frontal sulcus (SFS-P) is located at the level of the caudal limit of the rostrum of the corpus callosum (Fig. 3F). Here, we identified a gradient regarding SPR-S orientation from the last common ancestor of humans and old-world monkeys to the last common ancestor of humans and apes. In hominids, it always displays a vertical orientation compared to SFS (i.e., running parallel to the dorsal part of the central sulcus), and, in old-world monkeys, it can also present such an orientation, although more rarely (*F* = 50.46, NumDF = 3, DenDF = 512.78, *P* < 2.2 × 10^−16^, GLMM; Fig. 3D). Note that, as previously demonstrated with regard to the medial prefrontal cortex (*12*), the presence of gradients of sulcal organization in the four species studied is a marker of homologies.

In human and chimpanzee brains, SPR-S joins the superior frontal sulcus in 99% of hemispheres (SFS-P in human and fs in chimpanzee; Fig. 3, B and C). Topographically, in old-world monkeys, there is a dimple located just anterior to the precentral dimple (*26*) that is likely to correspond to the human SFS-P. First, its frequency of occurrence displays a gradient in the primate order (*F* = 101.18, NumDF = 3, DenDF = 506.08, *P* < 2.2 × 10^−16^, GLMM; Fig. 3C). Second, when assessing the configuration of this dimple, it appears to join the precentral dimple in old-world monkeys, more frequently in baboons (in 19% of hemispheres) than in macaques (in 4% of hemispheres), strongly indicative of a correspondence with SFS-P (Fig. 3E).

Furthermore, in old-world monkeys, two additional dimples are systematically observed dorsal to the principal sulcus and rostral to PMFS-P. The assessment of their location reveals that the caudal and rostral dimples are respectively located at the level of the rostral limit of the genu of the corpus callosum and of the supra-rostral (SUROS)/sus-orbitalis (SOS) junction located at the rostral-most part of the cingulate sulcus (CGS) (fig. S3). In human brains, the caudal and rostral limits of anterior superior frontal sulcus (SFS-A) are also found at the same locations, respectively (fig. S8). In chimpanzees, the segmented fs suggests that it corresponds to SFS-P and SFS-A. In support of this hypothesis, we observed that the caudal and rostral limits of SFS-A in chimpanzees are located at the same anatomical levels as in human brains. Together, these data strongly suggest that the old-world monkey homolog of SFS-A is the two dimples described in this study and that the chimpanzee homolog of SFS-A is the anterior part of the sulcus previously referred to as fs (Fig. 1B).

### Hominid correspondence of the principal sulcus in old-world monkeys

**Fig. 4.**
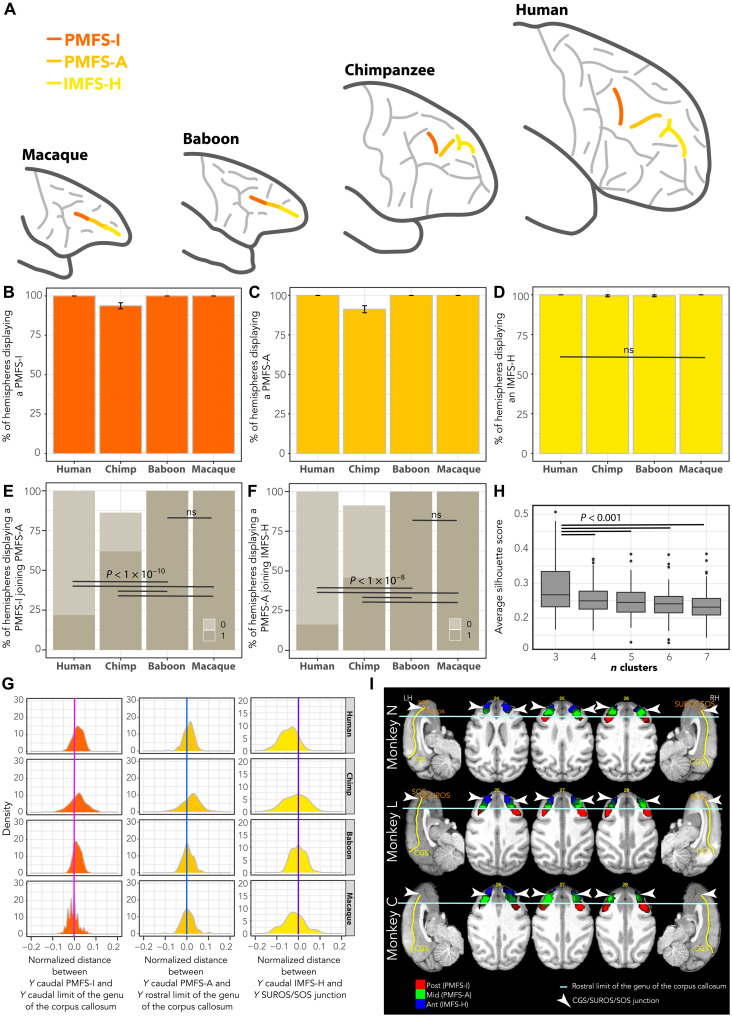
Hominid correspondence to the principal sulcus in old-world monkeys. (**A**) Identification and location of the sulci forming the principal sulcus in old-world monkeys and their homologs in hominids. Frequency of occurrence of PMFS-I (**B**), PMFS-A (**C**), and IMFS-H (**D**). (**E**) Percentage of hemispheres in which PMFS-I joins PMFS-A. (**F**) Percentage of hemispheres in which PMFS-A joins IMFS-H. (**G**) Normalized distance between the (i) *Y* level of the caudal-most part of PMFS-I and the *Y* level of the rostral limit of the caudal limit of the genu of the corpus callosum (dark orange), (ii) *Y* level of the caudal-most part of PMFS-A and the *Y* level of the rostral limit of the genu of the corpus callosum (light orange), and (iii) *Y* level of the caudal-most part of IMFS-H and the *Y* level of the intersection between the CGS, SUROS, and SOS (yellow). (**H**) Average silhouette score across the two hemispheres of the three macaques performing 12 rs-fMRI runs (see Materials and Methods). The best number of clusters is 3. (**I**) Probability maps of these three clusters (Post, Mid, and Ant) across 12 runs for each macaque (N, L, and C). The borders of the three functional clusters correspond to anatomical landmarks, strongly suggesting that the posterior (post), middle (mid), and anterior (ant) clusters correspond to, respectively, PMFS-I, PMFS-A, and IMFS-H. GLMM and/or Tukey post hoc tests at *P* < 0.05 (see Materials and Methods). LH, left hemisphere; RH, right hemisphere.

A major finding of the present study is that the principal sulcus of macaques and baboons and the fm in chimpanzees are not linear single sulci as previously considered. They are instead composed of distinct segments corresponding to known sulci in the human brain (Tables 1 and 2 and Fig. 4). Morphological features of nonlinearity are most notable within the depth of the sulcus but are sometimes apparent on the cortical surface (see examples in fig. S9). Three joining sulcal segments could be identified in old-world monkeys. Those segments are not contiguous in >20% of hemispheres in chimpanzees and in >50% of hemispheres in humans (Fig. 4, E and F). The extents of the caudal-most segment and of the rostral-most segment were located at the level of the caudal and rostral limits of the genu of the corpus callosum and at the level of the junction between the CGS and SUROS/SOS junction, respectively. At these anatomical levels, we identified the caudal-most parts of intermediate posteromedial frontal sulcus (PMFS-I), anterior posteromedial frontal sulcus (PMFS-A), and horizontal ramus of the intermediate frontal sulcus (IMFS-H) in humans and in chimpanzees (Fig. 4G). Furthermore, the frequency of occurrence of the three sulci is highly preserved across species (Fig. 4, B to D).

The existence of subdivisions along the principal sulcus is further supported by cytoarchitectonic differences of cortex of the dorsal bank of the principal sulcus in macaques. Changes at the interface between layer II and III could be observed along the rostro-caudal axis (fig. S10). Examination of connectivity with retrograde tracers also revealed heterogeneity of connections along the rostro-caudal axis (*27*, *28*). The scarcity of functional evidence to provide further support for this view is mainly due to the focus of most electrophysiological investigations on the caudal half of the principal sulcus (fig. S11). Nevertheless, functional heterogeneity has been reported in the rare studies that have specifically addressed this question (*29*–*31*). Beyond the heterogeneity of area 46 in macaques, the existence of additional subdivisions along the principal sulcus is supported by awake rs-fMRI analysis in macaque monkeys (see Materials and Methods). Data-driven connectivity-based parcellation (see Materials and Methods) indicated that the optimal number of parcels is 3 (Fig. 4H). The borders of the parcels in both hemispheres did also correspond to the position of the morphological nonlinearity in the sulcus, corresponding to the junction between PMFS-I and PMFS-A and to the junction between PMFS-A and IMFS-H (Fig. 4I). Petrides and Pandya (*21*) have shown that three cytoarchitectonic areas lie along the caudo-rostral axis of the principal sulcus: area 9/46, area 46, and area 10. In human brains, respective homolog areas lie in PMFS-I, PMFS-A, and IMFS-H, the rostral-most part of this sulcus being located in area 10 (fig. S6), reflecting the expansion of this area in apes and humans compared to old-world monkeys.

**Fig. 5.**
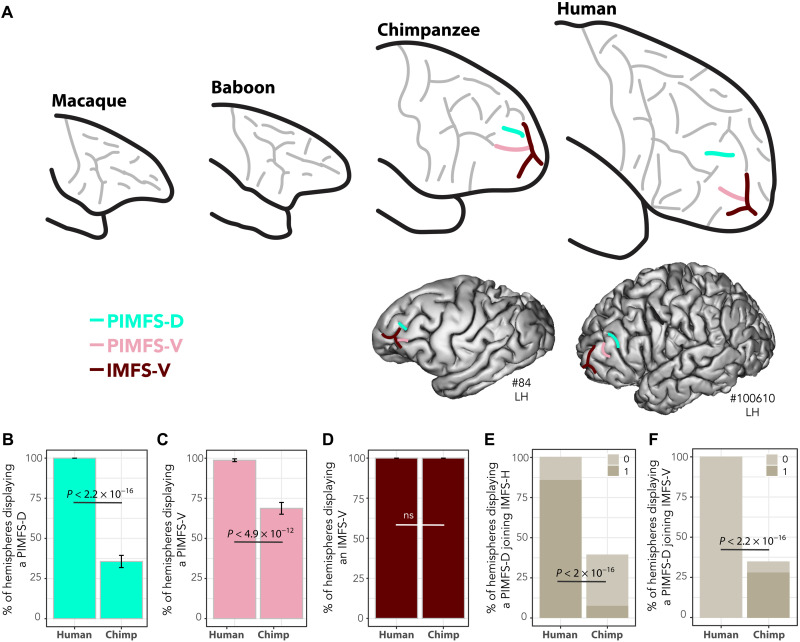
Hominid-specific sulci. (**A**) Human correspondence of PIMFS-D, PIMFS-V, and IMFS-V in chimpanzees. (**B**) Frequency of occurrence of PIMFS-D is decreased in chimpanzees (*F* = 225.85, NumDF = 1, DenDF = 158, *P* < 2.2 × 10^−16^). (**C**) Frequency of occurrence of PIMFS-V is decreased in chimpanzees (*F* = 55.9, NumDF = 1, DenDF = 158, *P* < 4.9 × 10^−12^, GLMM). (**D**) Frequency of occurrence of IMFS-V is equal in humans and chimpanzees (ns at *P* < 0.05, GLMM). (**E**) PIMFS-D joins more frequently IMFS-H in humans than in chimpanzees (*F* = 138.46, NumDF = 1, DenDF = 141.72, *P* < 2.2 × 10^−16^, GLMM). (**F**) PIMFS-D joins more frequently IMFS-V in chimpanzees and never in humans (*F* = 38.75, NumDF = 1, DenDF = 214, *P* < 2.51 × 10^−9^, GLMM). GLMM and/or Tukey post hoc tests at *P* < 0.05 (see Materials and Methods).

Alternative interpretations of the identity of the rostral-most segment of the principal sulcus are unlikely. First, IMFS-H is the only sulcus in the rostral part of the frontal cortex and spatially close enough to PMFS-A, which is highly preserved in chimpanzees. Second, dorsal paraintermediate frontal sulcus (PIMFS-D) and ventral paraintermediate frontal sulcus (PIMFS-V) display a drop in frequency of occurrence compared to humans (Fig. 5, A to C). Third, vertical ramus of the intermediate frontal sulcus (IMFS-V) is also present in chimpanzee and human brains (Fig. 5D) but never joins PMFS-A in these two species. Rather, only PIMFS-D joins either IMFS-H (in human; Fig. 5E) or IMFS-V (in chimpanzee, Fig. 5F). Last, correspondence with the inferior and superior frontal sulci was excluded as candidates for the principal sulcus.

## DISCUSSION

By combining sulcal pattern analysis with cytoarchitectonic analysis and rs-fMRI, the present study provides updated insights into the evolution of the frontal lobe across primates. Despite apparent discrepancies in the number of sulci, the lateral frontal lobe has a comparable organization across the primate order, complementing similar conclusions reached about the medial frontal cortex (*12*, *32*, *33*). Regardless of the phylogenic distance between primate species (*34*), we observed an apparent relationship between brain size, gyrification, and similarity to human sulcal organization (Tables 1 and 2 and Fig. 1D). Only three sulci appeared in hominids reflecting the expansion of the rostral prefrontal cortex (areas 10, 46, and 9): IMFS-V, PIMFS-D, and PIMFS-V. In all the frontal regions assessed in the present report, none appeared unique to the human brain. Despite suggestions that the lateral frontopolar cortex is uniquely human (*32*, *35*, *36*), the detailed analysis of sulcal morphology provides clear evidence for a common evolutionary history in hominids, with the lateral frontal cortex presenting a similar topological organization in human and chimpanzee brains.

The present study provides important clarifications about the macaque to human comparison of the frontal cortical organization. First, our results strongly suggest that PMFS-P does correspond to the border between the prefrontal and the premotor cortex, both in macaque and human brains. In macaque brains, it has been repeatedly shown that the dorsal part of the arcuate sulcus (i.e., PMFS-P in the human-based nomenclature; see Table 1) limits cytoarchitectonic area 6 from area 8 (fig. S6) (*21*). In human brains, although a formal cytoarchitectonic study should be conducted to provide a state-of-the-art demonstration, PMFS-P has also been suggested as being the limit between the frontal and the prefrontal cortex based on its functional connectivity profile (*37*). Second, a most notable and established functional aspect of the genu of the arcuate sulcus in macaque monkeys is the localization of the frontal eye field (FEF) in the anterior bank of the arcuate sulcus (*38*), i.e., at the intersection between PMFS-P and IPRS-S. By contrast, in humans, the FEF has largely been described as being located at the intersection between the SPRS and SFS-P (*3*, *5*, *39*). Although these two FEFs have often been viewed as homologous (*3*, *5*, *39*), the present results support an alternative interpretation. Whereas only one FEF has been reported in macaques, two FEF have been observed in humans: a dorsal FEF, i.e., the most commonly described (see above), and a ventral one (called iFEF and located in IPRS-S), largely neglected so far (*7*, *8*, *39*). The present study points toward homologies between the macaque FEF and the human iFEF, in line with a recent hypothesis (*20*). Clarifying this critical question would require additional studies assessing the respective connectivity and function of FEF and iFEF in the human brain and identifying their homologs in macaques [for example, with spider matching techniques; (*40*)]. Last, our results emphasize that the principal sulcus does not correspond to the IFS, as previously suggested (*41*). Rather, we show that the anatomical and functional organization of the principal sulcus lies entirely in the dorsolateral prefrontal cortex, in line with the known cytoarchitectonic organization of this region in which areas 9/46 and 46 occupy both banks of the principal sulcus and expand ventrally to the inferior frontal dimple (*21*, *42*).

A strong and highly reliable empirical result shown in the present and past studies (*12*, *37*, *43*–*45*) is the preserved topology of the sulcal organization of the cerebral cortex within and across primate species. Although the mechanisms supporting this conserved cortical folding pattern are not yet fully understood, such preservation is expected given the common developmental origin of the cortical areas in the primate order. First, it has been demonstrated that the cytoarchitectonic organization of the frontal cortex is topologically preserved in macaques and humans (*20*). Specifically, the frontal cortex is composed of several areas distinguished by their particular laminar organization, which display the same overall spatial location within and across primate species. The cortical laminar differentiation appears to be under the strong influence of genetic factors and governed by specific forces. Genetic factors appear to induce differential speeds of cell proliferation, thus creating heterogeneities in the ventricular zone that determine the endpoints of cortical neurons and the size of the cortical area [protomap hypothesis; (*46*)]. Then, the dual origin theory, originally developed by Sanides (*40*, *47*), suggests that the cerebral cortex is formed during development through two progressive sequences of laminar differentiation emerging from two distinct cortical progenitors: a ventral one, the pyriform cortex, and a dorsal one, the indusium griseum. In the frontal cortex, the sequence running from the pyriform cortex induces ventral-to-lateral forces responsible for the formation of the orbitofrontal and ventrolateral cortex, whereas the one running from the induseum griseum induces dorsal-to-lateral forces responsible for the formation of the medial and dorsolateral cortex. Second, the cortical folding appears later during development and seems also governed by several factors (*46*, *48*). Genetic influences, rather than pure external forces, seem to have a critical role in the formation of sulci and may explain the sulcal topology (*46*). The sulcal pits, i.e., the first location where a given sulcus develops (*42*), thought to relate closely to cytoarchitectonic areas (*46*, *49*), have been shown to be under a strong genetic control (*50*–*52*) exerted regionally (*53*). By contrast, models suggesting that the sulcal formation is governed by sole mechanistic, geometric constraints (*54*–*56*) do not explain the extremely well-preserved topological organization of sulci across hemispheres, individuals, and primate species. Third, probably as a result of the two previous points, cortical sulci and cytoarchitectonic areas appear to display tight relationships. Although this point is still debated, we argue that the only proper way to assess this particular aspect is to section the brain perpendicular to the direction of each sulcus of interest to have access to the optimal laminar organization of the cortex located on each side of a particular sulcus (*57*). Studies assessing specifically the relationships between sulci and cytoarchitectonic areas have revealed that sulci are either limiting or axial to cytoarchitectonic areas (*57*). For instance, (i) the central sulcus is the limiting sulcus between the primary motor cortex (area 4) and the primary somatosensory cortex (area 3) both in macaques and humans (*58*–*60*), (ii) the inferior frontal dimple is the limiting sulcus between area 45 and area 9/46 (*20*, *21*), and (iii) the CGS is limiting between area 24c′ and 32′ (*61*–*63*). Last, the cerebral cortex expands in the primate order around highly preserved subcortical structures, and we have shown in the present and past studies (*12*) that the landmarks of these subcortical structures (see fig. S3) are proxies for the topological organization of the sulcal cortical patterns. As the genome is highly similar across primate species (*64*, *65*), one can thus hypothesize that identical genetic influences and forces may constrain locally the sulcal expansion in all primates species and lead to conserved topological sulcal organization in the primate order. As such, the cortical expansion observed in the primate order should not result in sulci forming in widely distant places. Although our analysis needs to be expanded to the entire cerebral cortex to provide a definitive answer to this question, our empirical findings to date confirm this hypothesis.

In addition to inference about brain evolution, sulcal pattern analysis is an important tool for investigation brain function. In the human brain, the sulcal morphology of the frontal cortex has been associated with interindividual variability in the localization of brain activity (*3*, *5*, *6*, *66*, *67*) and in behavioral performances (*68*–*70*). Combined with the present study, these results suggest that the between-species interindividual sulcal cortical variability may be further interpreted in relation to the evolution of cognition. Specifically, whereas a preserved organization of the sulcal organization of a given cortical region across species may suggest that precursors of the abilities supported by this region were already present in the last common ancestors of hominids and old-world monkeys, a differential organization may suggest the emergence or the improvement of a given function.

To conclude, our work demonstrates the existence in macaque brains of the precursors of all the major sulci in the posterior frontal, dorsolateral, and frontopolar cortex of hominids, providing critical evidence for the value of the macaque primate brain in experimental, anatomical, and physiological neuroscience targeted to these regions. We expect that the present framework will allow a more accurate transfer of information from the nonhuman primate brain to the human brain.

## MATERIALS AND METHODS

Neuroimaging T1 anatomical data of 80 human, 80 chimpanzee, 80 baboon, and 80 macaque brains were analyzed. Note that the source of the neuroimaging data and most of the procedures are described in details in our previous publication (*12*).

### Anatomical neuroimaging data in human subjects

High-resolution anatomical scans of the human brain were obtained from the Human Connectome Project database (humanconnectome.org) (*71*). Acquisition parameters of T1 anatomical scans are the following: whole head, 0.7-mm^3^ isotropic resolution, TR (repetition time) = 2.4 s, TE (echo time) = 2.14 ms, and flip angle = 8° (details can be found on https://humanconnectome.org/storage/app/media/documentation/s1200/HCP_S1200_Release_Appendix_I.pdf). The full set of inclusion and exclusion criteria is detailed elsewhere (*71*). The experiments were performed in accordance with relevant guidelines and regulations, and all experimental protocols were approved by the Institutional Review Board (IRB) (IRB no. 201204036; Title: “Mapping the Human Connectome: Structure, Function, and Heritability”). All subjects provided written informed consent on forms approved by the IRB of Washington University in St. Louis.

### Anatomical neuroimaging data in nonhuman primates

High-resolution anatomical scans of chimpanzee and baboon brains were obtained from the laboratories of W.D.H. and A.M., respectively. High-resolution anatomical scans of macaque brains were obtained from the laboratories of E.P., C.A., W.D.H., J.S., F.H.-B., and S.B.H. These data are now available in the PRIME-DE database (*72*–*74*). Data collected initially for studies on macaque monkeys and baboons were conducted under local ethics agreements [licenses from the U.K. Home Office; Provence and Lyon ethics committees] and in accordance with The Animals (Scientific Procedures) Act 1986 and with the European Union guidelines (EU Directive 2010/63/EU). Chimpanzee data collection was approved by the Institutional Animal Care and Use Committees at Yerkes National Primate Research Center (YNPRC) and University of Texas MD Anderson Cancer Center (UTMDACC) and also followed the guidelines of the Institute of Medicine on the use of chimpanzees in research.

### Anatomical neuroimaging data analysis across species

Human and macaque brains were normalized in the human (www.bic.mni.mcgill.ca/ServicesAtlases/HomePage) and macaque (*75*) MNI steretotaxic coordinate systems, respectively. Chimpanzee and baboon brains were normalized in the chimpanzee (*76*) and the baboon (*77*) standard brain, respectively. Note that normalization of all primate brains consisted in linear registrations, which allow within-species comparisons between brains without altering relationships between sulci and gyri. As previously indicated (*12*), it is thus unlikely that such processing influences commonality and divergence of sulcal organization observed between species. Normalization of primate brains was performed with SPM12 (www.fil.ion.ucl.ac.uk/spm/software/spm12/) (*12*).

We examined the organization of sulci of the lateral frontal cortex in the entire dataset (80/160 brains/hemispheres for each species). This analysis consisted in the identification of the probability of occurrence of all sulci in the lateral frontal cortex and in the assessment of any relationships that may exist between these sulci and certain fixed anatomical landmarks across the species examined (see fig. S3 for description of these landmarks). Specifically, we assessed whether important characteristics of sulci (i.e., the caudal limit of SPRS-S, IPRS-S, IPRS-P, IFS, PMFS-P, PMFS-I, PMFS-A, and IMFS-H and the rostral limit of SFS-P, IFS, and PMFS-P) were located at the level of specific anatomical landmarks (i.e., rostral limit of the mammillary body, caudal limit of the rostrum, anterior commissure, rostral limit of the optic chiasma, caudal and rostral limit of the genu of the corpus callosum, and junction between the CGS and the fork composed of the SOS and the SUROS sulcus; see fig. S3). Toward that goal, we calculated the differences between the *Y* value of these sulcal characteristics and the *Y* value of the various anatomical landmarks. Note that we did not have any specific hypothesis regarding the spatial relationship between a given anatomical landmark and a particular sulcal characteristic. Following procedures previously described by Amiez *et al.* (*12*), these differences were calculated in all four species on the normalized T1 data and then normalized to take into account the different anteroposterior extent of the brains of the four species (i.e., 175, 110, 85, and 60 mm, respectively, in the human, chimpanzee, baboon, and macaque brains). The normalization performed within species was obtained by dividing the *Y* coordinate of the sulcus of interest, measured on brains registered linearly in the species-specific standard space, by the anteroposterior extent of the standard brain. For a given sulcal characteristic (e.g., the caudal-most part of PMFS-P), we calculated, in all individuals of each specie, the normalized difference between the anteroposterior level (*Y* coordinate) of this characteristic with the anteroposterior level (*Y* coordinate) of each anatomical landmarks used in the present study (fig. S3). The sulcal characteristic associated with the median the closest to 0 was assigned as being located at the level of this anatomical landmark. The add-on of baboon data is of considerable interest (although this species belongs to old-world monkeys) because the baboon brain is bigger and more gyrified than the macaque brain, and, as shown in (*12*), the slightly more gyrified brain of the baboon exhibits a tendency toward the hominid sulcal configuration in comparison with the macaque brain that is slightly less gyrified. The sulcus labeling was performed by C.A., and difficult cases were discussed with J.S. and M.P., as described by Amiez *et al.* (*12*).

We tested the influence of species on the probability of occurrence of a sulcus with logistic regressions. In the statistical models, “species” (human, chimpanzee, baboon, and macaque) was the independent variable, and “presence” (0, 1) of a sulcus was the dependent variable. To assess whether the probability of observing the various sulci was similar or different in each species, we performed GLMM in each species with these sulci as independent variable and the subject ID as random factor, i.e., formula: sulcus presence ~ species + (1 | ID). *F*, *P*, NumDF, and DenDF values from the GLMM are reported. Post hoc Tukey tests were then applied. These data are presented in dataset S1. All statistics were performed with R software, R Development Core Team (*78*) under R Studio (*79*).

### rs-fMRI experiment in awake macaques

The aim was to identify any subdivisions of the principal sulcus in macaques using a data-driven approach, blind from local morphological variability in the sulcus. Because we have recently shown that the frontal cortical connectivity is highly affected by anesthesia (*80*), we restricted our analysis of rs-fMRI data obtained in awake macaques.

#### Data acquisition

Three rhesus monkeys (*Macaca mulatta*) were included in the study (two females: monkeys C, 21 years old, and N, 9.5 years old; one male: monkey L, 9.5 years old; weight, 5 to 8 kg). Animals were maintained on a water and food regulation schedule, individually tailored to maintain a stable level of performance for each monkey. All procedures follow the guidelines of European Community on animal care (European Community Council, directive no. 86-609, 24 November 1986) and were approved by French Animal Experimentation Ethics Committee no. 42 (CELYNE). Scanning was performed on a 3T Siemens Magnetom Prisma MRI Scanner (Siemens Healthcare, Germany). All detailed information about awake monkey training, surgery, and experimental rs-fMRI setup can be found in (*80*). The rs-fMRI acquisition parameters were the following: TR = 1800 ms, TE = 27 ms, flip angle = 75°, field of view (FOV) = 480 mm by 336 mm, voxel size = 1.8 mm isotropic, and 30 slices. For each macaque, 12 runs of 400 volumes were acquired.

#### Data analysis

Preprocessing steps were carried out using MATLAB toolbox SPM12, AFNI software [Analysis of Functional NeuroImages; (*81*)], and FSL software [FMRIB Software Library; (*82*)]. The preprocessing procedure is described in detail in (*80*). Both anatomical and functional images were registered in a common atlas space, i.e., the CHARM atlas [https://afni.nimh.nih.gov/pub/dist/doc/htmldoc/nonhuman/macaque_tempatl/atlas_charm.html; (*83*, *84*)], to ensure optimized intersession and intersubject comparisons.

All rs-fMRI processing steps were realized with AFNI software. A temporal filtering was applied to extract the spontaneous slowly fluctuating brain activity (0.01 to 0.1 Hz). Linear regression was used to remove nuisance variables (the six parameter estimates for head motion, the cerebrospinal fluid, and white matter signals from the segmentation). Last, a spatial smoothing with a 4-mm full-width half maximum Gaussian kernel was applied to the output of the regression.

We then performed a data-driven parcellation of the principal sulcus. The first step was to identify and manually draw a mask covering the entire principal sulcus, separately in the left and right hemispheres of each macaque on the basis of the anatomical MRI scan. Visualization and drawing were performed with AFNI software. Each resulting principal sulcus mask was resampled from anatomical (voxel size = 0.5 mm^3^) to functional dimensions (voxel size = 1.8 mm isotropic). We computed, in each hemisphere, each macaque, and each run, Pearson’s correlation (*r*) between the time course of each voxel of the principal sulcus mask and the time course of each voxel composing the gray matter of the whole brain. These *r* values were then transformed to *z* scores. The output was, for each subject, *n* three-dimensional (3D) matrix with *n* being the number of voxels in the principal sulcus mask. Each 3D matrix was flattened to reach an *n*-by-*m* 2D matrix representing *z* scores between each voxel of the principal sulcus mask (*n* rows) and each voxel of the rest of the gray matter of entire brain (*m* columns).

On the basis of previous work (*85*, *86*), we applied a clustering algorithm to this matrix to classify voxels depending on their *z* score profile with voxels composing the gray matter of the entire brain. Clustering algorithms aim at joining elements displaying the same features into the same group, element with dissimilarities being included into other groups. Thus, we expected that voxels of the principal sulcus mask displaying similar connectivity profiles would be clustered together and would correspond to a particular principal sulcal subregion associated with a particular whole-brain connectivity profile. We applied spectral clustering algorithms because of their reliance on graph theory, which is known to reflect well brain connectivity (*87*). From the previous 2D matrix representing the whole-brain connectivity profile of each voxel of the principal sulcus mask, we computed the adjacency matrix measured by the correlation between rows. We then used the *k*-nearest neighbor to extract the similarity matrix. From this latter matrix, the Laplacian matrix and its spectral decomposition were computed, and we applied the *K*-means algorithm on the eigenvalues matrix to obtain the clusters (*88*). Results showed that highly connected voxels in the principal sulcus mask were grouped together. All clustering procedures were built with Python 3.8.10 and following libraries: scikit-learn (*89*) for all the clustering part, pandas (*90*) for the data management, numpy (*91*) for calculation and matrix building and operation, and nibabel (*92*) for NiFti file management.

To identify the optimal number of clusters composing each principal sulcal mask based on a pure data-driven approach, we used silhouette index scores. This score measures the ratio of the sum of between-cluster and within-cluster dispersions for all clusters (where dispersion is defined as the sum of distances squared) and is higher when clusters are dense and well separated. We tested the influence of the number of parcels on silhouette index scores across macaques with mixed general linear model GLMMs in which “number of parcels” (1➔7) was the independent variable, “silhouette index scores” was the dependent variable, and subject and run ID are random factors. Post hoc Tukey tests were then applied. The resulting optimal number of clusters (*n* = 3, see results) was then used to parcellate the CGS mask of each hemisphere and in each macaque and each run. Last, for each macaque and each hemisphere, a probability map over the 12 runs was computed. These data are presented in dataset S2. All statistics were performed with R software, R Development Core Team under R Studio (*79*).

#### Cytoarchitectonic analysis of the dorsal principal sulcus in macaques

Three brains were initially perfused with saline solution followed by a formalin solution to ensure the fixation of the tissue. Before sectioning the tissue using cryotome, the entire brain was placed in a phosphate-buffered saline 20% sucrose solution. The brain was then sectioned along the anterior-posterior axis, with consecutive 40-μm slices processed for different staining protocols and with one of the six sections taken for cresyl violet staining. Sections were then scanned using an AxioScan, Leica. Qualitative analyses of obtained images were then conducted by J.S. and V.M.-L. Results are presented in fig. S10.

#### Meta-analysis of electrophysiological studies

A PubMed search revealed 211 recording studies published between 1990 and 2020 on the dorsolateral or dorsal prefrontal cortex in monkeys. Of those studies, only 67 were included in the meta-analysis because they provided numerical values along the anterior-to-posterior axis of the actual recording sites or the localization of the recording chamber. If data from an animal were included in several studies, then only one study was considered. One study (*93*) was excluded from the analysis because of the large discrepancy (>10 mm) between the single-subject coordinates and the atlas used for reference (*94*). Figure S11 reports the distribution of recording sites in the principal sulcus (and adjacent dorsal region) of the dorsolateral prefrontal cortex in macaque monkeys. Studies included in the meta-analysis are provided in the Supplementary Materials.

## Notes

View/request a protocol for this paper from *Bio-protocol*.
