# Supplementary material for: A revised perspective on the evolution of the lateral frontal cortex in primates
Source: Sci Adv. 2023 May 19;9(20):eadf9445. doi: 10.1126/sciadv.adf9445 (PMC10198639; doi:10.1126/sciadv.adf9445)
Supplement: Supplementary file 1 — Figs. S1 to S12 Legends for data S1 and S2 [file sciadv.adf9445_sm.pdf]

Science Advances
AAAS

Supplementary Materials for

A revised perspective on the evolution of the lateral frontal cortex in primates

Céline Amiez *et al.*

Corresponding author: Céline Amiez, celine.amiez@inserm.fr

Sci. Adv. **9**, eadf9445 (2023)
DOI: 10.1126/sciadv.adf9445

The PDF file includes:

Figs. S1 to S12
Legends for data S1 and S2

Other Supplementary Material for this manuscript includes the following:

Data S1 and S2

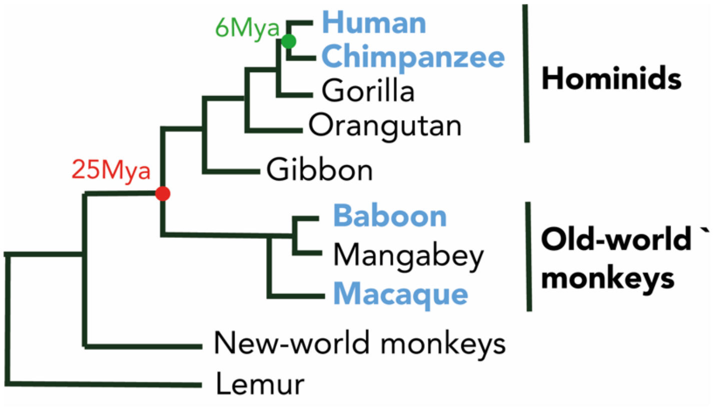

Fig. S1. Phylogenetic tree. The studied species are indicated in blue. Abbreviations: Mya, Millions years-ago.

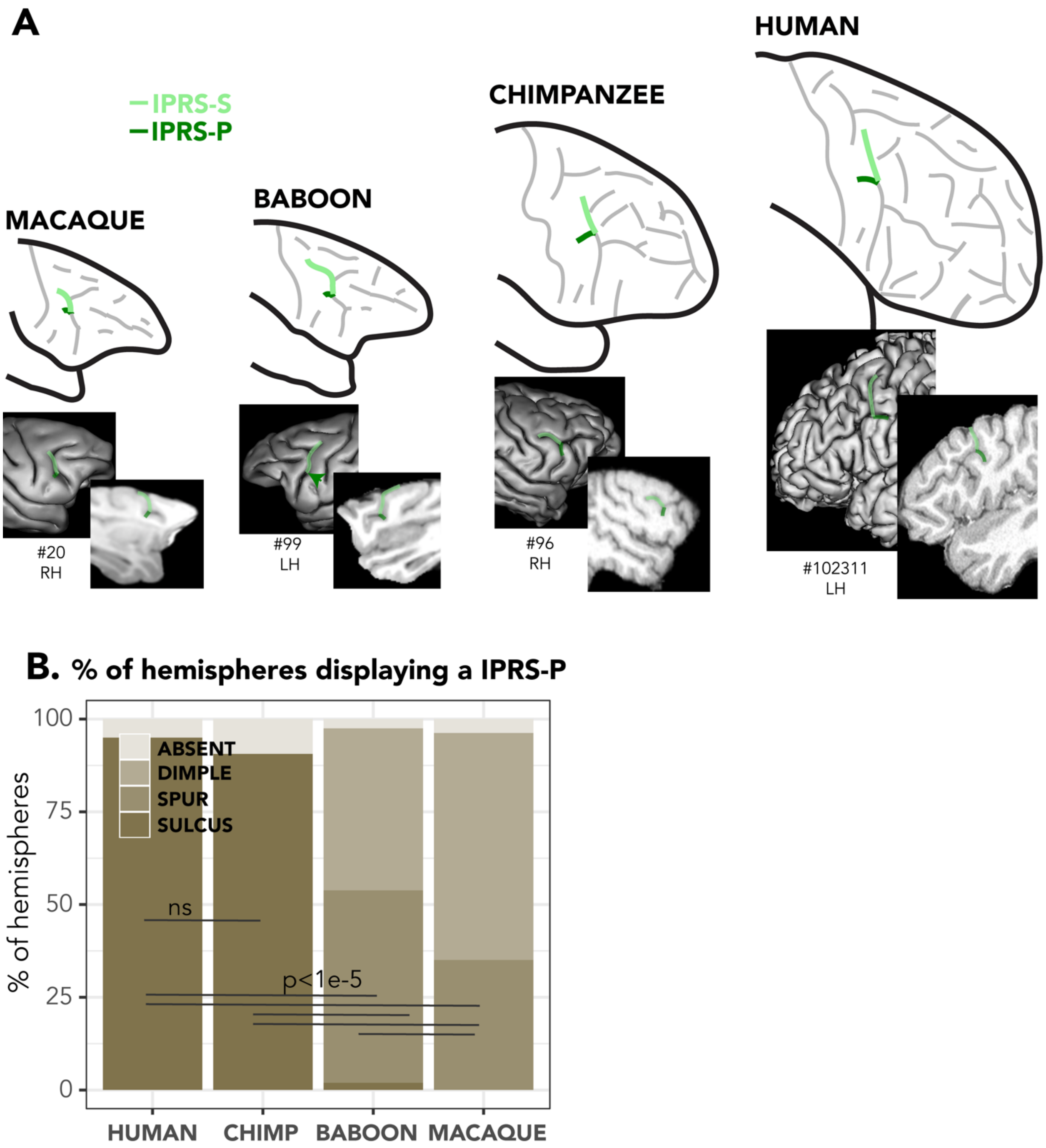

Fig. S2. IPRS-S and IPRS-P correspondence between the 4 species. (A) Example of IPRS-S and IPRS-P sulci on cortical surfaces and sagittal views of typical primate brains. **(B)** Frequency of occurrence of hemispheres in which IPRS-P is a dimple, a spur, and a sulcus. IPRS-P is a sulcus in both humans and chimpanzees, more often a spur in baboon, and more often a dimple in macaque (at p<1.e-5, GLMM). Abbreviations: ns, non-significant; LH, RH, left and right hemispheres.

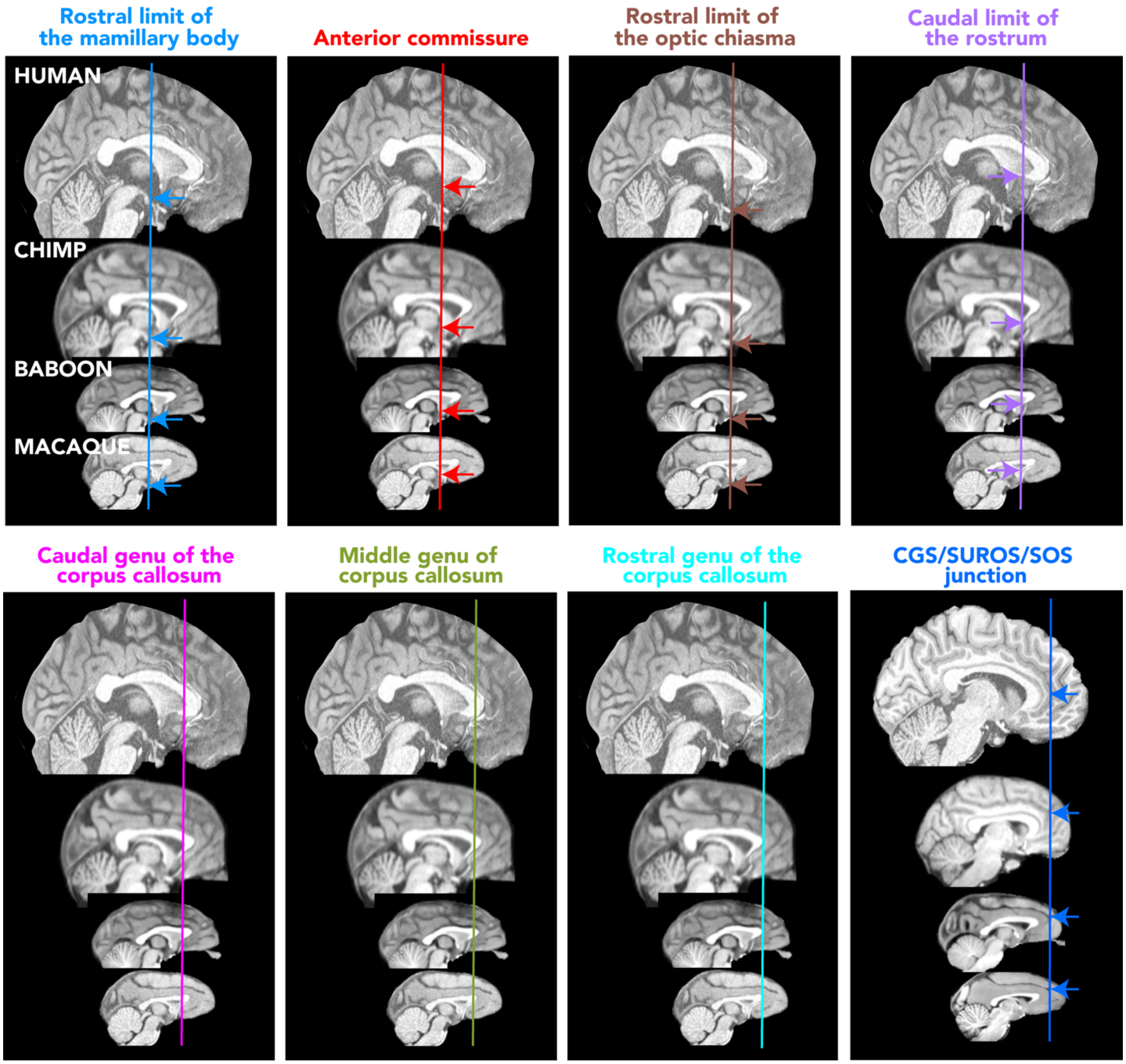

Fig. S3. Postero-anterior levels of all anatomical landmarks studied in the 4 species. From posterior to anterior: 1) the rostral limit of the mamillary body, 2) the anterior commissure, 3) the rostral limit of the optic chiasma, 4) the caudalmost part of the rostrum, 5) caudalmost, 6) middle, 7) rostralmost part of the corpus callosum, and 8) the junction of the cingulate sulcus (CGS) with the fork composed by the suprarostral (SUROS) and the sus-orbitalis (SOS) sulcus in the medialsurface of the brain.

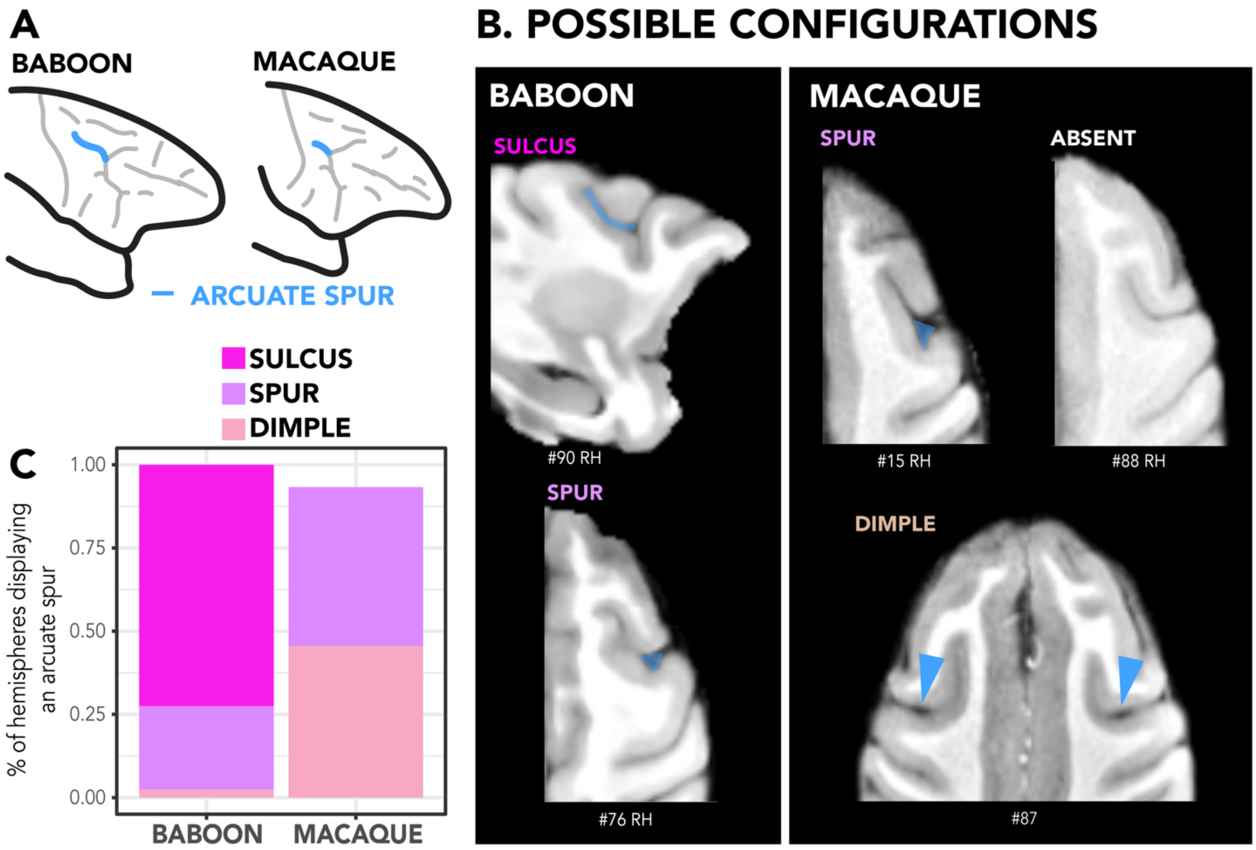

Fig. S4. Configuration and frequency of occurrence of the arcuate spur. (A) Location of the arcuate spur in old-world monkeys. **(B)** Possible configurations of the arcuate spur in baboon (sulcus or spur) and macaques (spur, dimple, absent). These configurations are shown on sagittal and horizontal slices on typical brains. **(C)** Frequency of occurrence of hemispheres displaying an arcuate spur in the configuration of a sulcus, spur, and dimple (pointed by the blue arrow), in baboon and macaque brains. The arcuate spur appears more frequently as a sulcus in the baboon brain, and equally as a spur or a dimple in the macaque brain. Abbreviations: LH and RH, left and right hemispheres.

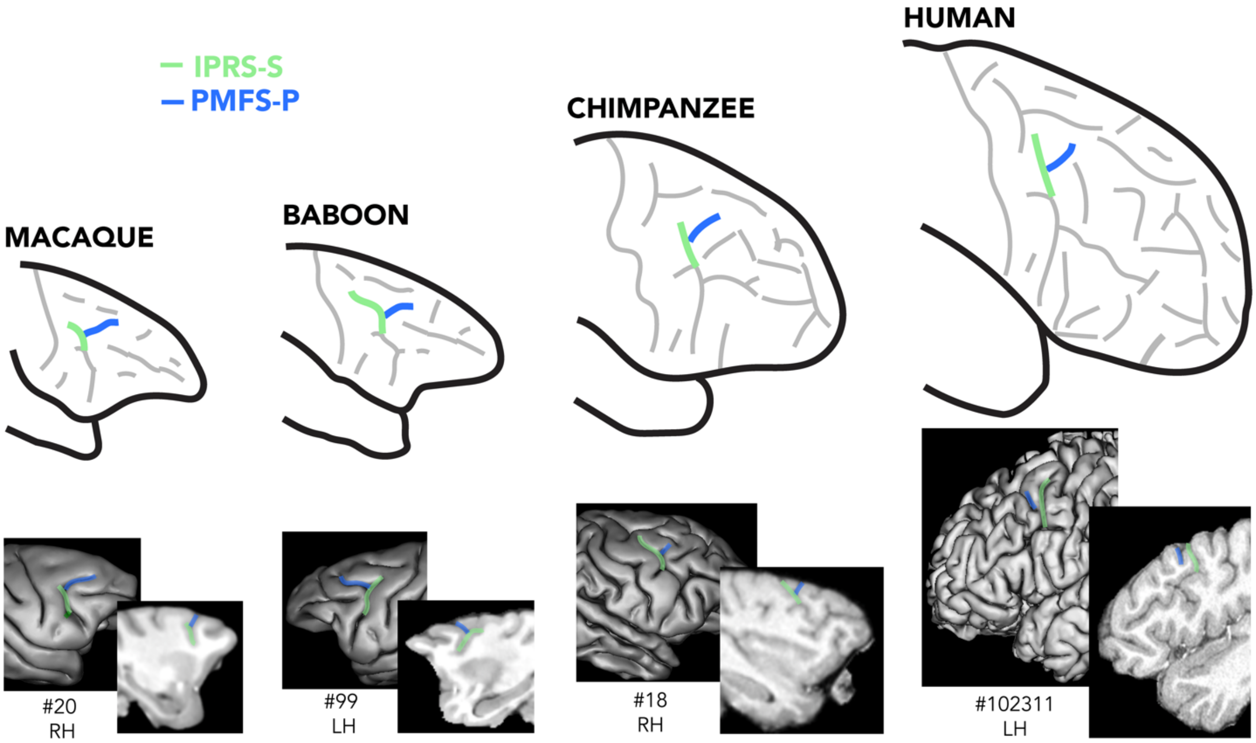

Fig. S5. Example of IPRS-S and PMFS-P sulci in cortical surface and sagtital views in typical primate brains. Abbreviations: LH, RH, left and right hemisphere.

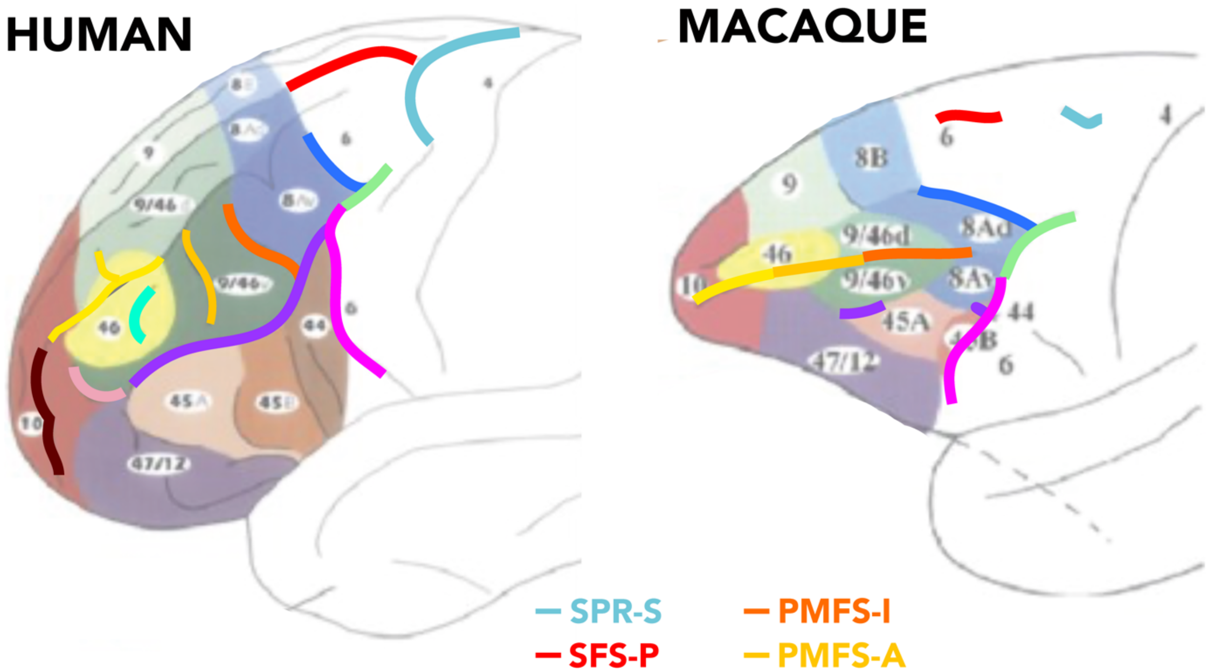

Fig. S6. Relationships between sulci organization in the human and macaque frontal cortex and known cytoarchitectonic organization from Petrides and Pandya (1994).

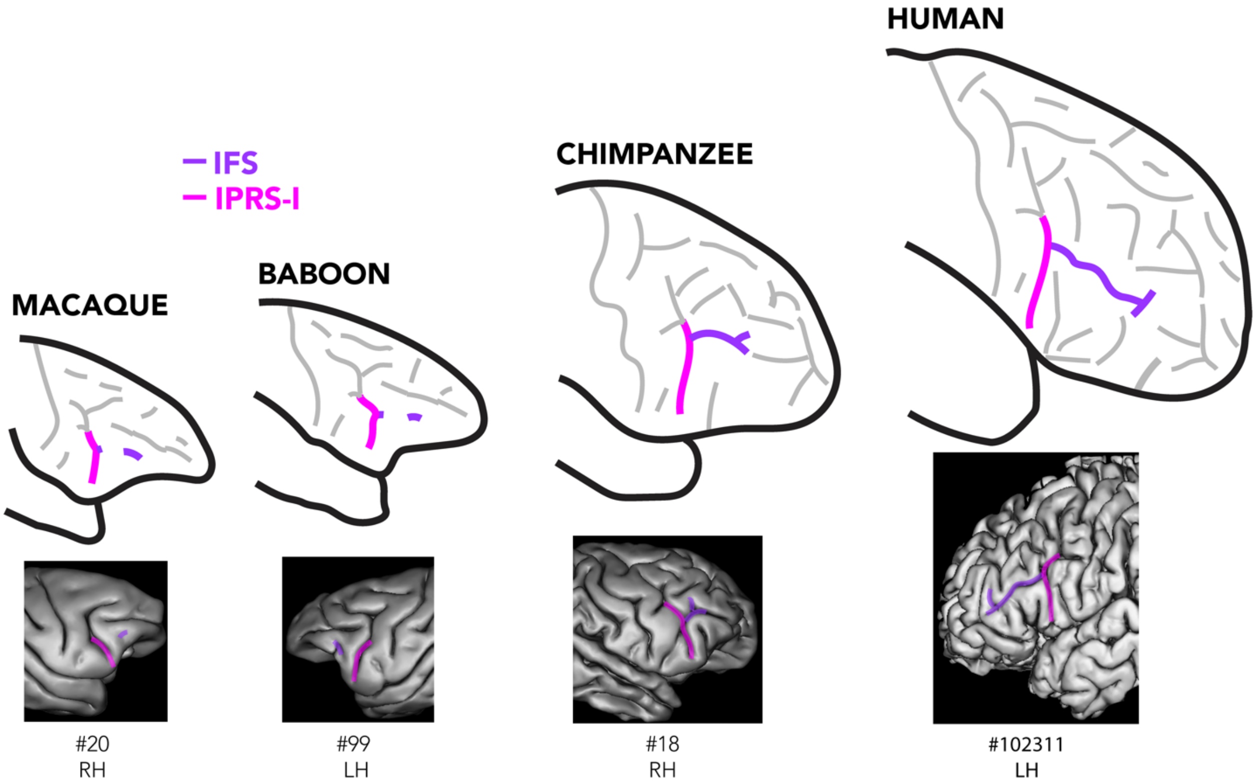

Fig. S7. Examples of IPRS-I/IFS configurations in typical primate brains. Abbreviations: LH, RH, left and right hemispheres.

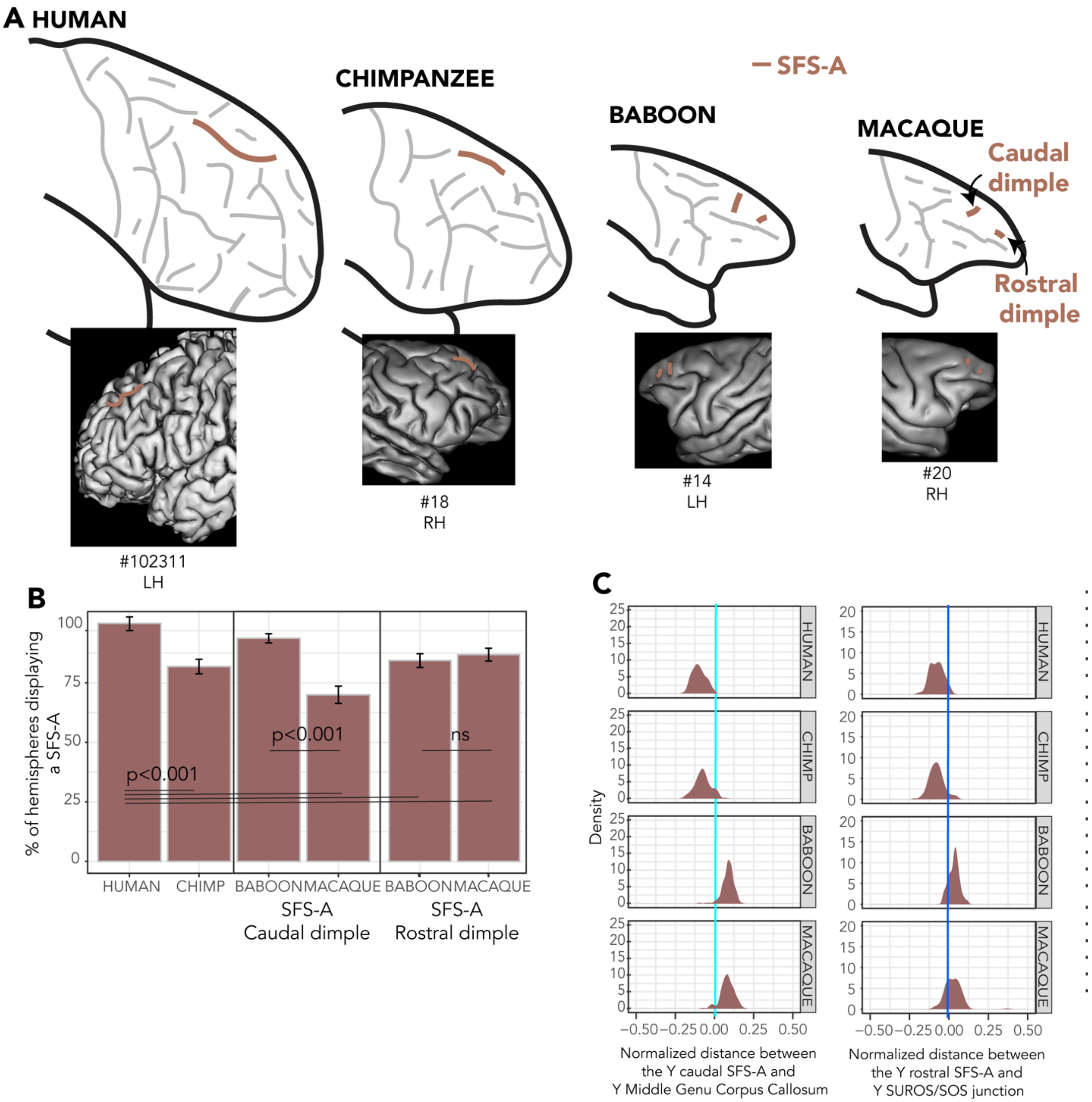

Fig. S8. Location and frequency of occurrence of SFS-A across the 4 species. (A) Hominids correspondence of SFS-A in old-world monkeys. In old-world monkeys, SFS-A is split in 2 segments, one forming the posterior part, and one forming the anterior part. **(B)** Frequency of occurence of SFS-A is decreased in non-human primates (F=25.92, NumDF=3, DenDF=508.93, p<1.29e-15, GLMM). **(C)** Normalized distance between the 1) Y level of the caudalmost part of SFS-A and the Y level of the middle part of the genu of the corpus callosum, and 2) Y level of the rostralmost part of SFS-A and the Y level of the caudal limit of the intersection observed in the medial surface between the rostralmost part of CGS with the fork formed by SUROS and SOS (see Fig S3). Abbreviations: ns, non-significant GLMM and/or Tukey post-hoc tests at p<0.05.

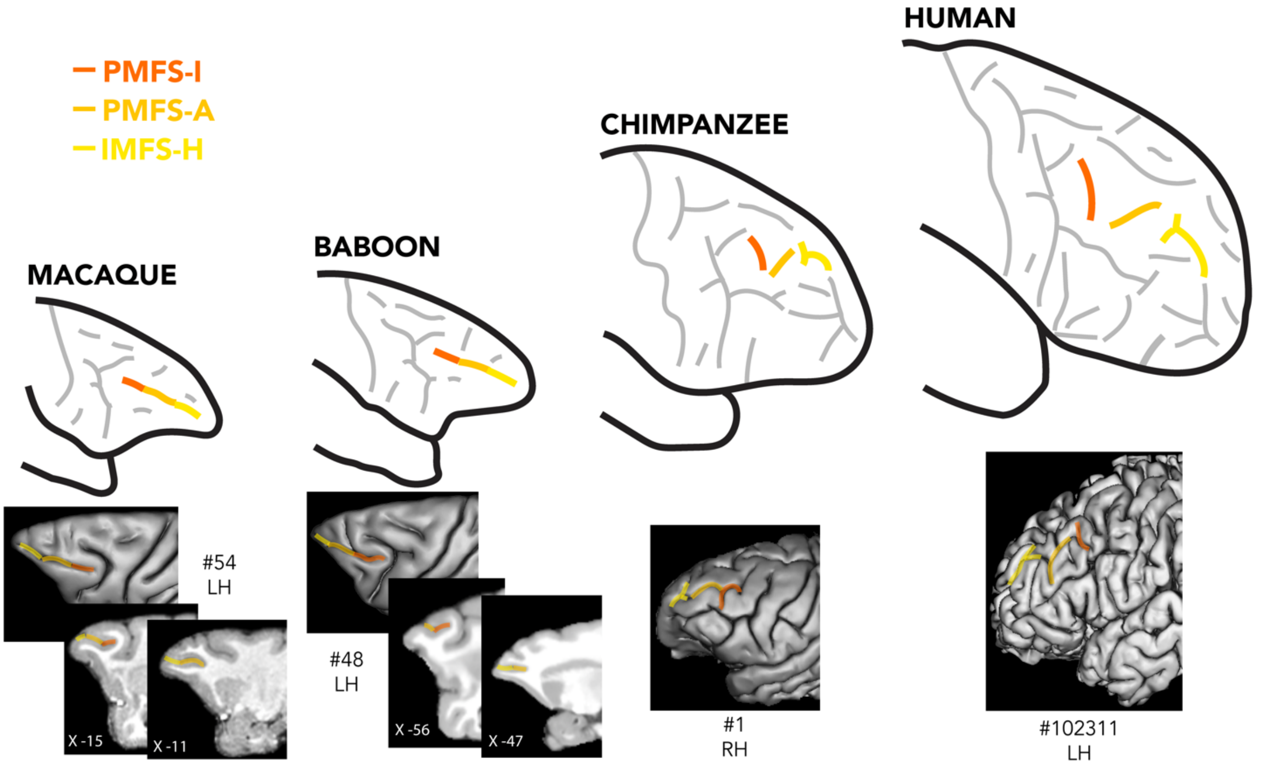

Fig. S9. Examples of typical subjects displaying A PMFS-I, PMFS-A, and IMFS-H sulci. Abbreviations: LH, RH, left and right hemispheres. X values indicate the medio-lateral level of the sagittal slices.

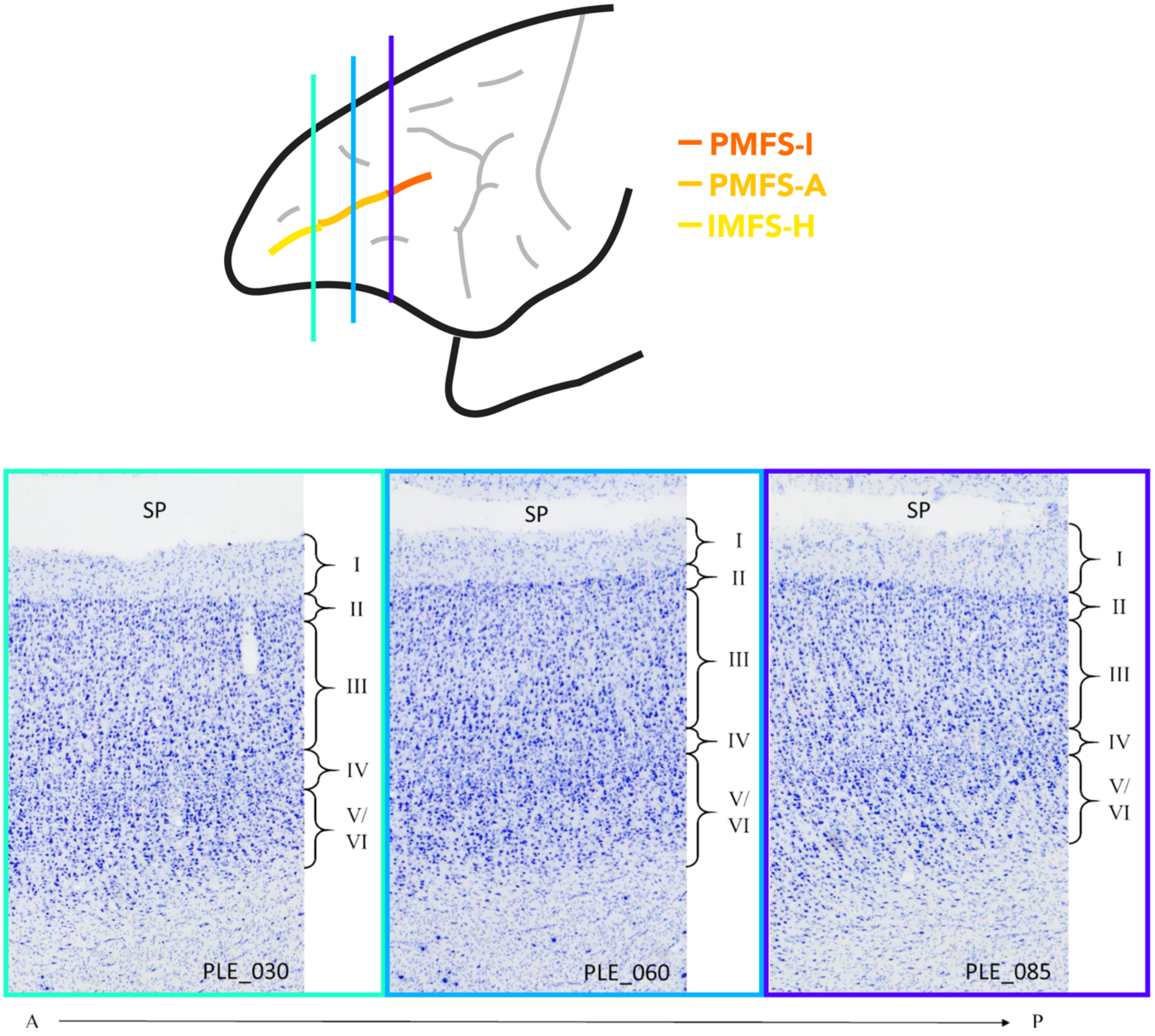

Fig. S10. Cytoarchitectonic organization of the principalis sulcus in macaque monkeys. Coronal slices of the dorsal bank of the principal sulcus at 3 different antero-posterior levels. Results show changes in transition between layer II and III along the rostro-caudal axis.

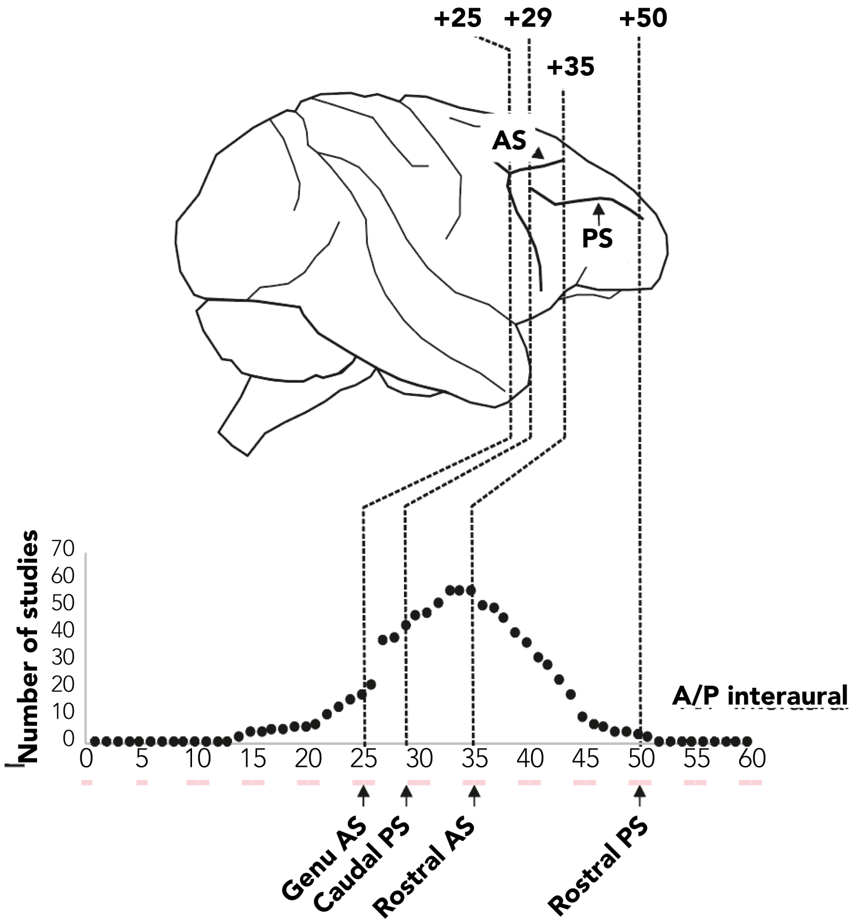

Fig. S11. Meta-analysis indicating the number of electrophysiological studies performed in the principal sulcus in macaque monkeys from 1990 to 2020 (i.e. references 95-140, see Dataset S2) and the antero-posterior level of the recordings compared to the antero-posterior level of the interaural line (A/P interaural). Most recordings are performed in the posterior part of the principal sulcus, preventing the complete understanding of the caudo-rostral anatomo-functional organization of the principal sulcus. Abbreviations: AS, PS, arcuate sulcus, PS, principal sulcus.

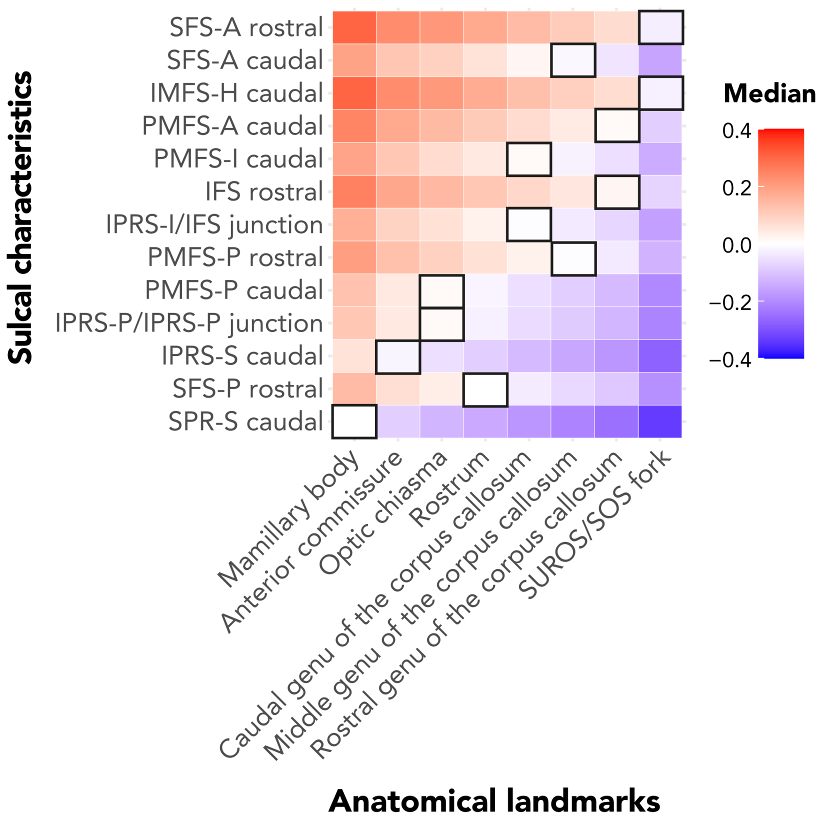

Fig. S12. Median across species of the normalized difference between the Y value of each sulcal characteristics (on Y axis, see Figs 2-5, S8) and the Y value of the various anatomical landmarks (on X axis, see Fig S3). For a given sulcal characteristic, the normalized difference with the various anatomical landmarks displaying the median the closest to 0 was assigned as being located at the level of this anatomical landmark (identified by black squares).

Data S1.

Data S1 provide, for each individual and each species, the coordinates used of all sulcal and anatomical landmarks at the source of the statistical analysis.

Data S2.

Data S2 provide the list of references included in the meta-analysis presented in Fig S11.
